# The sensitivity of network statistics to incomplete electrode sampling on intracranial EEG

**DOI:** 10.1162/netn_a_00131

**Published:** 2020-05-01

**Authors:** Erin C. Conrad, John M. Bernabei, Lohith G. Kini, Preya Shah, Fadi Mikhail, Ammar Kheder, Russell T. Shinohara, Kathryn A. Davis, Danielle S. Bassett, Brian Litt

**Affiliations:** Department of Neurology, Hospital of the University of Pennsylvania, Philadelphia, PA, USA; Center for Neuroengineering and Therapeutics, University of Pennsylvania, Philadelphia, PA, USA; Center for Neuroengineering and Therapeutics, University of Pennsylvania, Philadelphia, PA, USA; Department of Bioengineering, School of Engineering and Applied Science, University of Pennsylvania, Philadelphia, PA, USA; Center for Neuroengineering and Therapeutics, University of Pennsylvania, Philadelphia, PA, USA; Department of Bioengineering, School of Engineering and Applied Science, University of Pennsylvania, Philadelphia, PA, USA; Center for Neuroengineering and Therapeutics, University of Pennsylvania, Philadelphia, PA, USA; Department of Bioengineering, School of Engineering and Applied Science, University of Pennsylvania, Philadelphia, PA, USA; Department of Neurology, Hospital of the University of Pennsylvania, Philadelphia, PA, USA; Center for Neuroengineering and Therapeutics, University of Pennsylvania, Philadelphia, PA, USA; Department of Neurology, Emory University, Atlanta, GA, USA; Department of Biostatistics, Epidemiology, and Informatics, University of Pennsylvania, Philadelphia, PA, USA; Penn Statistics in Imaging and Visualization Center, University of Pennsylvania, Philadelphia, PA, USA; Center for Biomedical Image Computing and Analytics, University of Pennsylvania, Philadelphia, PA, USA; Department of Neurology, Hospital of the University of Pennsylvania, Philadelphia, PA, USA; Center for Neuroengineering and Therapeutics, University of Pennsylvania, Philadelphia, PA, USA; Department of Neurology, Hospital of the University of Pennsylvania, Philadelphia, PA, USA; Department of Bioengineering, School of Engineering and Applied Science, University of Pennsylvania, Philadelphia, PA, USA; Department of Electrical and Systems Engineering, School of Engineering and Applied Science, University of Pennsylvania, Philadelphia, PA, USA; Department of Physics and Astronomy, College of Arts and Sciences, University of Pennsylvania, Philadelphia, PA, USA; Department of Psychiatry, Hospital of the University of Pennsylvania, Philadelphia, PA, USA; Department of Neurology, Hospital of the University of Pennsylvania, Philadelphia, PA, USA; Center for Neuroengineering and Therapeutics, University of Pennsylvania, Philadelphia, PA, USA; Department of Bioengineering, School of Engineering and Applied Science, University of Pennsylvania, Philadelphia, PA, USA; Department of Neurosurgery, Hospital of the University of Pennsylvania, Philadelphia, PA, USA

**Keywords:** Intracranial EEG, Electrocorticography, Network model, Graph theory, Epilepsy, Reliability, Jackknife subsampling

## Abstract

Network neuroscience applied to epilepsy holds promise to map pathological networks, localize seizure generators, and inform targeted interventions to control seizures. However, incomplete sampling of the epileptic brain because of sparse placement of intracranial electrodes may affect model results. In this study, we evaluate the sensitivity of several published network measures to incomplete spatial sampling and propose an algorithm using network subsampling to determine confidence in model results. We retrospectively evaluated intracranial EEG data from 28 patients implanted with grid, strip, and depth electrodes during evaluation for epilepsy surgery. We recalculated global and local network metrics after randomly and systematically removing subsets of intracranial EEG electrode contacts. We found that sensitivity to incomplete sampling varied significantly across network metrics. This sensitivity was largely independent of whether seizure onset zone contacts were targeted or spared from removal. We present an algorithm using random subsampling to compute patient-specific confidence intervals for network localizations. Our findings highlight the difference in robustness between commonly used network metrics and provide tools to assess confidence in intracranial network localization. We present these techniques as an important step toward translating personalized network models of seizures into rigorous, quantitative approaches to invasive therapy.

## Author Summary

Network neuroscience applied to epileptic brains seeks to identify pathological neural connections that promote and maintain seizures, and holds promise to guide surgical planning in patients with intractable epilepsy. However, sampling of the epileptic network in intracranial EEG recording is limited by the choice of where to place intracranial electrodes, which is highly variable within and between epilepsy centers. The effect of incomplete spatial sampling generated by sparse electrode placement on network statistics is unknown. Here, we determine the sensitivity of several network statistics to incomplete spatial sampling, and we propose a method using electrode subsampling to determine patient-specific confidence intervals in network model predictions.

## INTRODUCTION

Epilepsy is a significant cause of disability worldwide, particularly among the one third of patients whose seizures cannot be controlled by medications (Kwan, Schachter, & Brodie, 2011; Wiebe, Eliasziw, Bellhouse, & Fallahay, 1999). While these patients may benefit from surgery or implanted devices, many continue to experience seizures after invasive therapies (Engel, 1996; Englot, Birk, & Chang, 2017; Noe et al., 2013; Wiebe, Blume, Girvin, & Eliasziw, 2001). One reason for this persistence of seizure activity may be the difficulty in localizing seizure-generating brain regions, the drivers of complex epileptic brain dynamics.

Clinicians and scientists now agree that epilepsy is in part a disease of brain networks (Kramer & Cash, 2012). Driven by clinical observations, scientists have applied formal models from network theory to better understand seizure dynamics and target therapy (Bassett, Zurn, & Gold, 2018). In these models, the brain is discretized into regions represented by network nodes, while network edges are used to represent their structural or functional connectivity. Network theory applied to epilepsy employs a wide variety of metrics to understand seizure generation and control, including node strength (Proix, Bartolomei, Guye, & Jirsa, 2017), eigenvector centrality (Burns et al., 2014), betweenness centrality (Wilke, Worrell, & He, 2011), clustering coefficient (Liao et al., 2010), and control centrality (Khambhati, Davis, Lucas, Litt, & Bassett, 2016; Kini et al., 2019), as well as global metrics including global efficiency (Pedersen, Omidvarnia, Walz, & Jackson, 2015), synchronizability (Khambhati et al., 2016), and transitivity (Paldino, Zhang, Chu, & Golriz, 2017). Collectively, these network measures have been used to predict neuronal firing as seizures begin and spread, track seizure progression, identify the seizure onset zone, and predict surgical outcome (Burns et al., 2014; Fletcher & Wennekers, 2018; Panzica, Varotto, Rotondi, Spreafico, & Franceschetti, 2013; Ponten, Bartolomei, & Stam, 2007; Sinha et al., 2017; Wilke et al., 2011).

When using invasive sensors such as intracranial EEG (iEEG) to estimate functional connectivity, sampling from the full brain is impossible, and the network measures available for modeling depend on the location and number of electrodes implanted. In fields outside of epilepsy, missing data are known to affect the results of network analyses (Albert, Albert, & Nakarado, 2004; Albert, Jeong, & Barabási, 2000; Guimerà & Sales-Pardo, 2009; Lü & Zhou, 2011). The effect of missing data on network models and clinical care in epilepsy has not been rigorously explored. While network models have potential to add rigor to clinical decision-making, their application may be limited by uncertainty in estimated network metrics and the unknown interaction between that uncertainty and sparse brain sampling. In this study we seek to rigorously assess the extent to which different network metrics are sensitive to intracranial electrode sampling. Our goal is not to determine which, if any, network statistic correctly localizes the seizure onset zone or predicts surgical outcome, as this important work is currently under way by several groups (Kini et al., 2019; Proix, Jirsa, Bartolomei, Guye, & Truccolo, 2018; Shah, Bernabei, et al., 2018; Sinha et al., 2017, 2019). Rather, our goal is to determine (a) whether and how incomplete spatial sampling affects the practical utility of network statistics, and (b) how sensitivity to spatial sampling can estimate patient-specific uncertainty in network model predictions. This computational work is a vital first step to deploying network models as an adjunct to clinical decision-making.

## MATERIALS AND METHODS

### Summary

We use a high-quality dataset that has been included in multiple network studies in epilepsy (Khambhati et al., 2015, 2016; Kini et al., 2019; Sinha et al., 2017) and is publicly available at www.IEEG.org. We randomly eliminate nodes from functional networks to simulate the uncertainty consequent to variable sampling of brain regions by iEEG and to determine the reliability of different network metrics within and across patients. Based upon the assumption that the main drivers of epilepsy network behavior might localize to an epileptogenic region, we ask to what extent electrode contacts far away from the seizure onset zone impact the estimated values of various network metrics, and whether subsampling that targets the seizure onset zone disproportionately affects network statistics compared with subsampling that spares the seizure onset zone. We then randomly remove nodes by jackknife subsampling in order to derive patient-specific estimates of confidence in network statistics.

### Patient Selection, Intracranial EEG Recording, and Electrode Localization

All patients gave written informed consent in accordance with the Institutional Review Board of the Hospital of the University of Pennsylvania (HUP) and the Mayo Clinic in Rochester. Furthermore, all patients consented to publishing their full-length iEEG recordings on the public web portal IEEG.org (Wagenaar, Brinkmann, Ives, Worrell, & Litt, 2013). This study was performed in accordance with the Declaration of Helsinki.

A total of 28 patients with drug-resistant epilepsy underwent iEEG recording during presurgical evaluation at HUP or the Mayo Clinic. Electrode configurations (Ad Tech Medical Instruments, Racine, WI) consisted of linear cortical strips and two-dimensional cortical grid arrays (2.3-mm diameter with 10-mm intercontact spacing), and linear depth electrodes (1.1-mm diameter with 10-mm intercontact spacing). EEG signals were recorded at a sampling frequency of 512 Hz at HUP and 500 Hz at Mayo Clinic. All electrode and EEG recording systems were FDA approved and are commercially available.

Each patient underwent MPRAGE T1-weighted magnetic resonance imaging (MRI) on a 3T Siemens Magnetom Trio scanner (Siemens, Erlangen, Germany) prior to electrode implantation, and they also underwent spiral CT imaging (Siemens, Erlangen, Germany) after electrode implantation. We cross-referenced the CT images with a surgical cartoon map to localize electrode coordinates (Wu et al., 2011). To segment the resection zone, we registered the preimplant MRI to postresection imaging and the postimplant CT using the Advanced Normalization Toolkit (ANTs; Avants et al., 2011). We utilized a random forest classifier with ITK-SNAP to semiautomatically estimate the resection zone and identify electrodes overlying resected cortex (Yushkevich et al., 2006).

Seizures were identified clinically and confirmed in a clinical case conference discussion. Board-certified epileptologists (Fadi Mikhail, Ammar Kheder, Kathryn Davis, and Brian Litt) then reviewed the seizures and identified the earliest electrographic change (EEC; Litt et al., 2001) and the electrode contacts of seizure onset (identified using the clinical standard for recognizing the electrode contact with the EEC) for each seizure. We performed our primary analysis on the first seizure identified for each patient. For patients with more than one seizure (*N* = 26), we also performed the analysis on the second seizure to assess the sensitivity of our results to the choice of seizure. For patients with three or more seizures (*N* = 23), we also performed the analysis on the patient’s last seizure in order to evaluate more temporally distant seizures, given evidence that temporally clustered seizures may have similar dynamics, and given the possibility that earlier seizures may be atypical because of postimplantation effect (Schroeder et al., 2019; Sun, Arcot Desai, Tcheng, & Morrell, 2018). One patient (HUP111) had two separate electrode implantations, and we analyzed both implantations separately.

### Calculating Functional Networks

We examined 1-s time windows (sampled at 512 Hz at HUP and 500 Hz at Mayo Clinic) at each of the following time periods: 10 s prior to the EEC, 5 s prior to the EEC, at the EEC, 5 s after the EEC, and 10 s after the EEC. We chose 1-s time windows so as to have sufficient data to perform coherence calculations and because of the validation of this time window in prior publications (Khambhati et al., 2016; Kini et al., 2019; Kramer et al., 2011). To determine the sensitivity of our results to this choice, we repeated this analysis with time windows of 2 s. We performed our primary analysis on the time period at the EEC given evidence for changes in network parameters that occur at the EEC (Khambhati et al., 2015, 2016). We then repeated the analysis for each other time window in order to assess the sensitivity of our results to the choice of time period, and given the evidence that both interictal networks and post-EEC networks localize the seizure onset zone (Burns et al., 2014; Shah, Bernabei, et al., 2018).

A common average reference was applied to iEEG signals to remove common sources of noise. Data were filtered using an elliptic bandpass filter with cutoff frequencies of 5 Hz and 115 Hz, as well as a 60-Hz notch filter to remove power line noise. Signals were prewhitened using a continuous autoregressive model to account for slow dynamics and to accentuate higher frequencies known to be involved in seizure dynamics. This also enhanced local neural population dynamics in order to minimize the effect of signal mixing (Arbabshirani et al., 2014; Khambhati et al., 2015; Towle, Carder, Khorasani, & Lindberg, 1999). For each 1-s window, we constructed functional networks in which iEEG electrode contacts represented network nodes. Edges were weighted by multitaper coherence, which estimates the correlation between two electrode contact signals in the frequency domain and is frequently used to calculate functional networks in neuroscience publications (Khambhati et al., 2016; Mitra & Pesaran, 1999; Weiss et al., 2015). We calculated coherence in the high gamma frequency band (95–105 Hz), which we chose because of its importance in seizure propagation and spread (Khambhati et al., 2016). We also repeated the analysis in beta (15–25 Hz) to assess the sensitivity of our results to the choice of frequency band, and in acknowledgment of the fact that the beta frequency is also thought to be important in epileptic networks (Bettus et al., 2011). This separation of the data resulted in an adjacency matrix for each frequency band representing a network with undirected, weighted edges for each patient, where each row and each column represented an electrode contact, and each matrix element represented the signal coherence between the two contacts.

To determine the sensitivity of our results to the choice of network density, we also performed weight-based thresholding in which we set matrix elements below a weight *w* to 0, where *w* was tuned for each patient to achieve a network density of 0.5 (in addition to the unthresholded network).

### Network Metrics

For each functional network, we calculated several global and nodal network metrics, chosen because of their importance in graph theory and their use in recent epilepsy publications as described above. The global metrics were synchronizability, global efficiency, and transitivity. The nodal metrics were node strength, control centrality, clustering coefficient, eigenvector centrality, and betweenness centrality. The methods for calculating these metrics have been previously described, and we briefly summarize each below. We specifically describe their calculations for an undirected, weighted network. We calculated each using the Brain Connectivity Toolbox (Rubinov & Sporns, 2010), or using custom code for synchronizability and control centrality (Khambhati et al., 2016).

#### Global metrics.

Global efficiency is a global measure that is thought to represent how easily information travels throughout the network (Latora & Marchiori, 2001). It is defined as

$$E=\frac{1}{N(N-1)}\sum_{i\neq j}\frac{1}{\sigma_{ij}},$$

where *E* is global efficiency, *N* is the number of nodes, and *σ*_*ij*_ is the shortest weighted path length between node *i* and node *j*, for example estimated using Dijkstra’s algorithm (Dijkstra, 1959). A high global efficiency is thought to reflect a greater ease of information transfer throughout the network (Bassett et al., 2009). Path lengths were weighted by the inverse of the values of the adjacency matrix, to reflect the fact that information is thought to be transferred more readily along stronger edges (Opsahl, Agneessens, & Skvoretz, 2010).

Synchronizability is a global metric that quantifies the stability of the fully synchronous network state (Boccaletti, Latora, Moreno, Chavez, & Hwang, 2006; Schindler, Bialonski, Horstmann, Elger, & Lehnertz, 2008) and has been shown to predict seizure generalization (Khambhati et al., 2016). It is calculated by first computing the weighted Laplacian *L* = *D* − *A* as the difference between the node strength matrix *D* and the adjacency matrix *A*. Synchronizability is then obtained by the equation *Sync* = $\frac{\lambda_{2}}{\lambda_{max}}$, where *Sync* is synchronizability, *λ*_2_ is the second smallest eigenvalue of the Laplacian, and *λ*_*max*_ is the largest eigenvalue. Greater synchronizability reflects a smaller spread between eigenvalues, which suggests greater ease for a network to synchronize its dynamics.

Transitivity is another global measure that represents the degree to which nodes in a graph tend to cluster together (Holland & Leinhardt, 1971; Opsahl & Panzarasa, 2009; Watts & Strogatz, 1998). It is defined as

$$T=\frac{\sum \tau_{\Delta }}{\sum \tau },$$

where *T* is transitivity, ∑*τ*_Δ_ is the sum of the weights of closed triplets, and ∑*τ* is the sum of the weights of all triplets. A triplet is defined as a set of three nodes connected by either two or three edges. A closed triplet, more specifically, is a set of three nodes connected by three edges. Higher transitivity implies that nodes tend to cluster together into exclusive groups.

#### Nodal metrics.

Node strength represents the total strength of connections involving a particular node (Fornito, Zalesky, & Bullmore, 2016), and is defined as

$$s_{i}=\sum_{j=1}^{N}A_{ij},$$

in which *s*_*i*_ is the strength of node *i*, *A*_*ij*_ is the adjacency matrix element containing the edge weight between node *j* and node *i*, and *N* is the number of nodes. A high node strength implies that the total weight of its connected edges is large. Eigenvector centrality is a similar nodal measure that weights individual node influence by the relative influence of each of its connected nodes (Fletcher & Wennekers, 2018; Newman, 2008). It is specifically defined as *λ* = *Ax*, where *x* is the vector containing the eigenvector centrality of each node, *A* is the adjacency matrix, and *λ* is the largest eigenvalue of the matrix (which results in nonnegative eigenvector centralities). A high eigenvector centrality implies that a node is strongly connected to nodes that themselves are highly connected.

Betweenness centrality is a nodal metric that is closely related to the global metric global efficiency and measures the fraction of all shortest paths in the network that pass through a given node (Freeman, 1977). It is defined as

$$b_{i}=\sum_{h\neq i\neq j}\frac{\sigma_{hj}(i)}{\sigma_{hj}},$$

where *b*_*i*_ is the betweenness centrality of node *i*, *σ*_*hj*_(*i*) is the number of shortest paths from node *h* to node *j* that pass through node *i*, and *σ*_*hj*_ is the total weighted path length between node *h* and node *j*. A high betweenness centrality suggests that the node acts as a central node in the shortest paths between many other nodes. The path lengths were weighted by the inverse of the values of the adjacency matrix as described above.

Control centrality is a local metric that measures the effect of each node on synchronizability. It is defined as *c*_*i*_ = $\frac{{Sync}_{new}-{Sync}_{old}}{{Sync}_{old}}$, where *c*_*i*_ is the control centrality of node *i*, *Sync*_*old*_ is the original synchronizability, and *Sync*_*new*_ is the synchronizability of the network with the node removed (Khambhati et al., 2016). Negative control centrality nodes are synchronizing, whereas positive control centrality nodes are desynchronizing.

Clustering coefficient is the nodal extension of transitivity that measures the amount of interaction between local triplets (Barrat, Barthélemy, & Vespignani, 2007). It is calculated by *cl*_*i*_ = 2∑_*k*,*j*_ $\frac{{\left(A_{ik}A_{ij}A_{kj}\right)}^{1/3}}{v(v-1)}$, in which *A* is the adjacency matrix edge weight and *v* is the number of neighbors. Higher clustering coefficients reflect greater clustering of the node into tight groups.

### Network Subsampling

**Figure 1.**
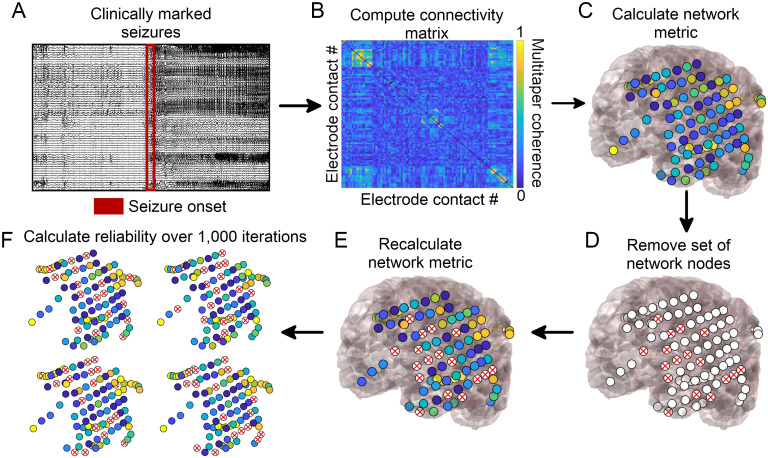
Network generation and subsampling methods. (A) Seizure onset times were marked by a board-certified epileptologist. (B) Multitaper coherence of a 1-s interval of EEG signal at seizure onset was used to create a functional adjacency matrix. (C) Network metrics were calculated using the adjacency matrix with all nodes included. (D) A subset of nodes were removed to simulate the effect of leaving out electrodes. (E) Network metrics were recalculated from the subsampled network. (F) This process was repeated over 1,000 iterations and the reliability of each metric was quantified.

To determine the sensitivity of network metrics to spatial sampling, we randomly identified electrode contacts for removal in each patient. We removed the rows and columns corresponding to these electrode contacts from the adjacency matrix representing the network. We recalculated each of the network metrics above. We performed this analysis removing 20%, 40%, 60%, and 80% of randomly selected electrode contacts. We repeated this process 1,000 times for each removal percentage to obtain a distribution of new metrics in the randomly subsampled network (Figure 1).

### Determining Metric Reliability

To determine the stability of each network metric to subsampling, we calculated the reliability for each removal percentage (Davidshofer & Murphy, 2005). Reliability is defined as *R* = $\frac{\sigma_{T}^{2}}{\sigma_{X}^{2}}$, where $\sigma_{X}^{2}$ = $\sigma_{T}^{2}$ + $\sigma_{E}^{2}$, and $\sigma_{T}^{2}$ is the variance of the true scores, $\sigma_{E}^{2}$ is the variance of the error, and $\sigma_{X}^{2}$ is the total variance. We defined the variance of the error to be the variance of the subsampled metric across the 1,000 random subsamples, averaged across electrode contacts in the case of nodal metrics. For nodal metrics, we defined the variance in the true scores to be the variance of the subsampled metric across electrode contacts, averaged over all permutations. In the case of global metrics, we defined the variance in the true scores to be the variance in the subsampled metric across patients, averaged over all permutations. Reliability is constrained to be between 0 and 1, where 1 indicates that no variance is due to random subsampling, 0 indicates that all variance is due to random subsampling, and 0.5 indicates that the variance due to random subsampling equals the variance of the true metric. The goal of the reliability measure is to compare how much metrics vary across subsamples relative to how much they vary across patients (in the case of global metrics) or electrode contacts within the patient (in the case of nodal metrics). Lower reliabilities suggest that the variance across subsamples is higher than that across patients (global metrics) or electrode contacts (nodal metrics), suggesting that minor changes in electrode configurations could result in different orderings of highest-to-lowest metric values, thus decreasing our confidence in the result. Of note, we calculated the variance in the true scores in the subsampled networks, rather than the original network, to avoid a bias in which some network metrics (such as node strength) have larger values and larger variances across electrodes in larger networks, resulting in paradoxically *higher* reliabilities when removing more electrodes.

To determine whether some metrics were more robust to subsampling than others, we compared the metric reliability across all patients for the 20% removal percentage using separate Friedman tests, one for global metrics and one for nodal metrics (*α* = 0.05; Friedman, 1937). In the case of significant Friedman test results, we performed post hoc Dunn-Šidák multiple comparisons tests to identify significant differences between individual metrics (Dunn, 1964; Šidák, 1967). We also determined the reliability of metrics for removal percentages other than 20%, which we report in our Supporting Information. We repeated this analysis for beta band coherence, alternate times relative to the EEC, removal of contiguous rather than random electrode contacts, alternate network densities, alternate time windows for calculating coherence, and different seizures, which we also report in our Supporting Information.

As an additional test of network stability to subsampling for nodal metrics, we calculated the Spearman rank correlation of the vector of nodal metrics across electrodes between the original network and each of the 1,000 subsampled metrics. We obtained the mean of the rank correlation across all 1,000 subsamples as a measure of the average correlation between the original set of nodal metrics and the subsampled metrics. We compared the metric rank correlations across all patients for the 20% removal percentage using a Friedman test (*α* = 0.05), performing post hoc Dunn-Šidák multiple comparisons tests to identify significant differences between individual metrics in the case of significant Friedman test results.

We then determined whether there was a relationship between the network reliability and the number of electrode contacts comprising the original network. We obtained the reliability for each patient and each nodal and global metric at the 20% removal percentage of random electrode contacts, using the EEC time period and gamma band coherence. For each metric, we correlated the reliability with the original number of electrode contacts in the patient’s network using Spearman rank correlation. We performed Bonferroni correction as we were testing eight network metrics, yielding an *α* of 0.05/8 = 0.00625.

### Influence of Seizure Onset Zone on Network Reliability

We next hypothesized that ictal network metrics may be more affected by removing electrode contacts near the seizure onset zone, as these contacts may have a stronger influence on epileptic networks. To test this, we again subsampled the network, this time systematically removing each electrode contact and its *N* − 1 nearest neighbors, where *N* was equal to 20% of the total number of contacts in the network (we also calculated it for other removal percentages and report these results in our Supporting Information). We recalculated each of the global and nodal metrics in this systematically subsampled network. We obtained a measure of agreement between the original metric and the new metric in the subsampled network. For nodal metrics, the agreement measure a was defined as the Spearman’s rank correlation coefficient across electrode contacts between the original and subsampled metric. For global metrics, the agreement measure was defined as the negative of the absolute value of the relative difference between the two metrics

$$a=-\left|\frac{{metric}_{new}-{metric}_{old}}{{metric}_{old}}\right|.$$

The global agreement *a* was equal to 0 when there was perfect agreement between the new and original metric, and was increasingly negative with larger absolute differences.

To test whether there was larger metric agreement when the removed electrode contacts were further from the seizure onset zone, we obtained the Spearman’s rank correlation coefficient between the agreement measure *a* with the distance between the centroid of the removed electrode contacts and the centroid of the seizure onset zone. We obtained the Fisher’s transformation of the rank coefficient for each patient, which is equal to the inverse hyperbolic tangent of the rank coefficient, in order to transform the coefficients to a variable whose distribution is approximately normal (Fisher, 1915). We aggregated these transformed rank coefficients across patients and performed a two-sided one-sample *t* test to determine whether the mean coefficient was significantly different from 0. We performed this test for each of the global and nodal metrics. We performed Bonferroni correction as we were testing eight network metrics, yielding an α of 0.05/8 = 0.00625.

As an additional test of the hypothesis that removing seizure onset zone electrodes disproportionately affects network statistics, we performed two additional subsampling methods: a seizure onset zone-targeted subsampling and a seizure onset zone-sparing subsampling. In the seizure onset zone-targeted subsampling, we identified all electrodes forming the clinician-defined seizure onset zone and we removed all of these and only these electrodes. In the seizure onset zone-sparing subsampling, we identified a randomly selected subset of electrodes, equal in number to the number of seizure onset zone electrodes, but excluding the seizure onset zone (in one patient, Study022, the number of seizure onset electrodes was more than half of the total number of electrodes, and in this case we removed all other electrodes for the seizure onset zone-sparing subsampling). We repeated the seizure onset zone-sparing subsampling 1,000 times. For each subsampling, we again calculated the agreement, *a*, between the original and subsampled network statistics, where *a* is defined above for both global and nodal metrics. We took the mean agreement across all 1,000 subsamples in the case of seizure onset zone-sparing subsampling. We compared the mean seizure onset zone-sparing agreement and the seizure onset zone-targeted agreement with a two-sided paired *t* test to determine whether the metric agreement when subsampling using a seizure onset zone-sparing method was significantly different from that using a seizure onset zone-targeted method. We performed this test for each of the global and nodal metrics. We performed Bonferroni correction as we tested eight network metrics, yielding an α of 0.05/8 = 0.00625.

As an alternative approach, we also calculated for each patient the percentage of seizure onset zone-sparing agreements that were higher than the seizure onset zone-targeted agreement. We performed a one-sample two-sided *t* test to determine whether the mean percentile was significantly different from 50% (under the null hypothesis that if the seizure onset zone contacts were not of particular importance to the network metrics, half of patients would be expected to have higher seizure onset zone-sparing versus seizure onset zone-targeting agreements), using a Bonferroni correction for eight network metrics (*α* = 0.00625). Of note, there were six patients for whom the number of seizure onset zone electrode contacts was large relative to the total number of electrode contacts (approaching half), and so for these patients there was likely a high interdependence between the 1,000 seizure onset zone-sparing subsamples. We expect that this makes this analysis less conservative than our primary analysis above.

We repeated the seizure onset zone analyses restricting analysis to patients with good (International League Against Epilepsy, ILAE = 1) outcomes (*N* = 13 patients), as it is possible that in the poor-outcome patients, the clinician-defined seizure onset zone did not accurately capture seizure generators. We also repeated these analyses using the electrodes overlying the resected area of cortex, rather than the seizure onset zone, while restricting analysis to ILAE 1 outcome patients, under the assumption that the resected cortex in these good-outcome patients likely overlaps with seizure generators.

### Deriving Patient-Specific Confidence in Network Results Using Jackknife Subsampling

We next utilized a jackknife subsampling method to generate patient-specific estimates in the confidence of the results of network analyses. Jackknife estimation is a method of sampling without replacement to derive statistical estimates (Quenouille, 1949, 1956; Tukey, 1958). It applies the same subsampling technique from our earlier analyses, but with the aim of obtaining patient-specific confidence rather than metric-specific reliabilities. Our goal was to determine how much a network result would be expected to change if a small number of electrode contacts had not been present. We randomly removed 20% of electrode contacts, recalculated the network statistic of interest for the random subsample, and repeated this process for 1,000 iterations. We chose a 20% removal percentage for this analysis to simulate minor variability in electrode implantation strategy. For each of the nodal metrics, we identified the electrode contact with the maximal metric value (minimal value for control centrality) in each of the 1,000 iterations. We identified the electrode contacts comprising 95% of all occurrences of the maximal metric value across the 1,000 iterations. We called this set of contacts the 95% jackknife confidence contact set. We also identified the 95% confidence contact set of the minimum *regional control centrality*, defined as the locations of an electrode contact and its *N* − 1 nearest neighbors, where *N* equals the number of resected electrode contacts that produces the largest negative change in synchronizability when removed. Regional control centrality attempts to identify a region of a defined size—rather than a single electrode contact—with the largest control centrality, and thus a potential site for resection (Kini et al., 2019). A larger 95% jackknife confidence set of electrode contacts implies greater sensitivity of the identity of the electrode contact with the maximal metric value to spatial subsampling, suggesting lower confidence in the patient-specific network result. For global metrics, we performed this method to obtain the 95% jackknife confidence interval for the value of the metric for a given patient, which was the interval containing 95% of all values obtained with jackknife subsampling. A larger 95% jackknife confidence interval for global metrics implies greater sensitivity of the global network statistic to spatial subsampling, suggesting lower confidence in the global network value. The runtime for the jackknife subsampling algorithm (1,000 iterations) for all metrics at a single time and frequency band was approximately ten minutes per patient when performed in MATLAB R2018a on an Intel Xeon processor (CPU E5-2698 v3 @ 2.30 GHz).

### Statistical Analysis

All analyses were performed on MATLAB R2018a (The MathWorks, Natick). Specific analyses are discussed in the four preceding sections, above.

## RESULTS

### Patient and Electrode Information

Patients had a variety of clinical histories, electrode configurations, pathologies, and clinical outcomes (Supplemental Table 1). There were 28 patients (13 women), one of whom had two temporally distinct implantations, which were separately analyzed. The average age at implantation was 33.9 years (range 5–57). The mean number of electrode contacts was 77 (range 16–118). The mean number of seizures was 6.8 (range 1–36). The median ILAE outcome score at 2 years was 2 (range 1–5).

### Stability of Metrics to Random Subsampling

**Figure 2.**
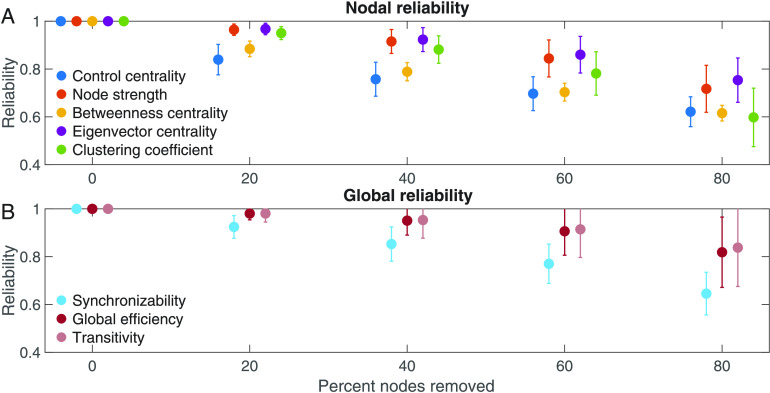
Reliability of network metrics to incomplete sampling. (A) Reliability of nodal measures, averaged across all patients, when different percentages of electrodes were removed. (B) Reliability of global measures, averaged across all patients, when different percentages of electrodes were removed. Error bars show the standard deviation of the reliability across patients. All data shown are for the EEC of first seizure, high gamma coherence, and random electrode removal. For all measures, reliability decreased as more electrodes were removed. Patients are heterogeneous in the reliability of their network measures, and certain network measures exhibit higher reliability than others.

For all network measures, reliability to subsampling decreased as more electrode contacts were removed. The stability of network measures to subsampling varied across patients (Figure 2). The mean reliability was *R* = 0.92 for synchronizability, *R* = 0.98 for global efficiency, and *R* = 0.98 for transitivity, averaged over all patients when a random sample of 20% of electrode contacts was removed. In contrast, when a contiguous sample of 20% of electrode contacts was removed, the mean reliabilities were lower, with *R* = 0.85 for synchronizability, *R* = 0.92 for global efficiency, and *R* = 0.93 for transitivity. The reliability to random electrode contact removal was significantly different between global metrics (Friedman test: $\chi_{2}^{2}$ = 36.5, *p* < 0.001). Synchronizability was significantly less reliable than either global efficiency or transitivity (post hoc Dunn-Šidák multiple comparison test: *t* = −3.02, *p* = 0.008 compared with global efficiency and *t* = −6.04, *p* < 0.001 compared with transitivity). Global efficiency was also significantly less reliable than transitivity (*t* = −3.02, *p* = 0.008). The reliability for global efficiency was slightly lower than that for transitivity for 26 out of 29 patient implantations. However, for two implantations the reliability for global efficiency was substantially larger, explaining why global efficiency and transitivity have similar means despite global efficiency having significantly lower reliability by ordinal statistics.

For nodal measures, the mean reliability was *R* = 0.84 for control centrality, *R* = 0.96 for node strength, *R* = 0.88 for betweenness centrality, *R* = 0.97 for eigenvector centrality, and *R* = 0.95 for clustering coefficient, averaged over all patients when 20% of electrode contacts were randomly removed (again, mean reliabilities were lower with contiguous removal, *R* = 0.82 for control centrality, *R* = 0.95 for node strength, *R* = 0.88 for betweenness centrality, *R* = 0.94 for eigenvector centrality, and *R* = 0.91 for clustering coefficient). The reliability differed significantly between nodal measures (Friedman test: $\chi_{4}^{2}$ = 107.9, *p* < 0.001). Control centrality was less reliable than node strength (post hoc Dunn-Šidák test: *t* = −6.73, *p* < 0.001), eigenvector centrality (*t* = −8.64, *p* < 0.001), and clustering coefficient (*t* = −3.99, *p* = 0.001) but was not significantly different from betweenness centrality (*t* = −1.00, *p* = 0.979). Node strength was also significantly more reliable than betweenness centrality (*t* = 5.73, *p* < 0.001) but did not differ significantly from eigenvector centrality (*t* = −1.91, *p* = 0.439) or clustering coefficient (*t* = 2.74, *p* = 0.060). Betweenness centrality was significantly less reliable than both eigenvector centrality (*t* = −7.64, *p* < 0.001) and clustering coefficient (*t* = −2.99, *p* = 0.028). Eigenvector centrality was more reliable than clustering coefficient (*t* = 4.65, *p* < 0.001).

When we examined the time periods 10 s before, 5 s before, 5 s after, and 10 s after the EEC (as opposed to the second at the EEC), synchronizability continued to have the lowest reliability of the global metrics. Control centrality continued to have the lowest reliability of the nodal metrics, and eigenvector centrality and node strength continued to have the highest reliabilities. The pattern remained when we examined beta frequency coherence rather than high gamma frequency coherence, when we removed contiguous as opposed to random sets of electrode contacts, when we examined the second seizure or the last seizure rather than the first seizure, when we thresholded the network weights to achieve a network density of 0.5, and when we used a 2-s time window for coherence calculations rather than a 1-s window (Supplemental Table 2). When we instead removed 40% or 60% of electrode contacts, control centrality and synchronizability continued to have the lowest reliability of nodal and global metrics, respectively, and node strength and eigenvector centrality continued to have the highest nodal metric reliabilities. When we removed 80% of electrode contacts, clustering coefficient instead demonstrated the lowest nodal metric reliability, and otherwise the pattern was unchanged (Supplemental Table 3).

For nodal metrics, the Spearman rank correlation between the original and subsampled met ric revealed the same ranking of metric robustness as with our primary approach. The mean Spearman rank correlation between the original metric and the subsampled metric, averaged across all subsamplings and all patients when 20% of electrode contacts were randomly removed, was ρ = 0.84 ± 0.08 for control centrality, ρ = 0.97 ± 0.02 for node strength, ρ = 0.91 ± 0.03 for betweenness centrality, ρ = 0.98 ± 0.02 for eigenvector centrality, and ρ = 0.97 ± 0.02 for clustering coefficient. The Spearman rank correlation significantly differed across nodal metrics (Friedman test: $\chi_{4}^{2}$ = 99.5, *p* < 0.001). The Spearman rank correlation between the original and subsampled control centrality metric was significantly lower than that for node strength (post hoc Dunn-Šidák test: *t* = −5.81, *p* < 0.001), eigenvector centrality (*t* = −8.64, *p* < 0.001), and clustering coefficient (*t* = −5.98, *p* < 0.001). The Spearman rank correlation between the original and subsampled node strength metric was significantly higher than that for betweenness centrality (*t* = 4.24, *p* < 0.001) and significantly lower than that for eigenvector centrality (*t* = −2.82, *p* = 0.046). The Spearman rank correlation between the original and subsampled betweenness centrality metric was significantly lower than that for eigenvector centrality (*t* = −7.06, *p* < 0.001) and for clustering coefficient (*t* = −4.40, *p* < 0.001). The comparisons between control centrality and betweenness centrality (*t* = −1.58, *p* = −0.704), node strength and clustering coefficient (*t* = −0.17, *p* = 1.00), and eigenvector centrality and clustering coefficient (*t* = 2.66, *p* = 0.076) were not significant.

We next examined the relationship between robustness to electrode contact subsampling and the number of electrode contacts in the original network. Among global measures, there was a significant positive relationship between reliability and number of contacts for synchronizability (Spearman rank correlation: *r*_27_ = 0.68, *p* < 0.001), but not for global efficiency (*r*_27_ = 0.20, *p* = 0.299) or transitivity (*r*_27_ = 0.22, *p* = 0.260). Among nodal measures, clustering coefficient (*r*_27_ = 0.53, *p* = 0.003) demonstrated a significant positive relationship, and relationships for control centrality (*r*_27_ = −0.12, *p* = 0.551), node strength (*r*_27_ = 0.45, *p* = 0.015), betweenness centrality (*r*_27_ = 0.38, *p* = 0.043), and eigenvector centrality (*r*_27_ = 0.44, *p* = 0.016) were nonsignificant (α = 0.00625, Bonferroni correction for eight measures). This pattern of findings suggests that, at least for synchronizability and clustering coefficient, patients with more electrode contacts implanted were less vulnerable to incomplete spatial sampling.

### Influence of Seizure Onset Zone on Sensitivity of Network Statistics to Subsampling

**Figure 3.**
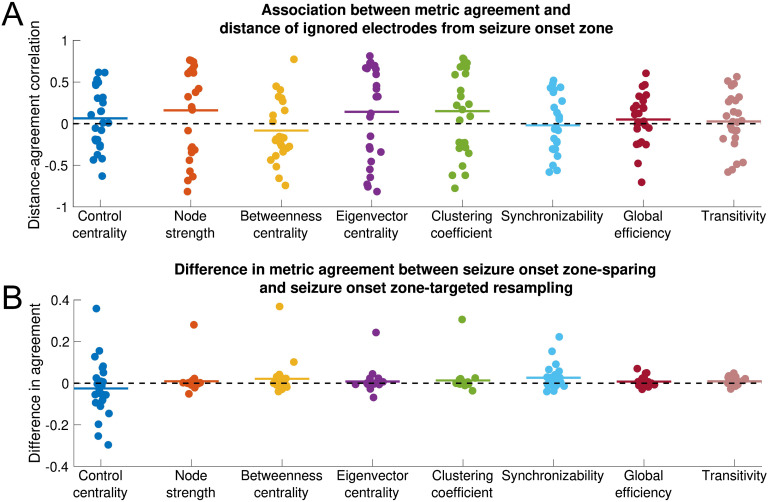
Influence of seizure onset zone on sensitivity of network statistics to subsampling. Each nodal and global metric is shown. All data shown are for the EEC of first seizure and high gamma coherence. (A) Association between metric agreement and distance of ignored electrode contacts from the seizure onset zone. Each point represents the patient-specific Spearman rank correlation coefficient between the metric agreement and the distance of the ignored electrodes from the seizure onset zone. The metric agreement is defined for nodal metrics as the Spearman rank correlation coefficient between the original metric and the metric obtained from subsampling, and for global metrics as the negative absolute value of the relative difference between the original and subsampled metric. Horizontal lines show the average distance-agreement association across patients. No distance-agreement association was significantly different from 0 (two-sided one-sample *t* test, α = 0.05/8 for Bonferroni correction), signifying that all metrics are equally vulnerable to incomplete sampling near versus distant from the seizure onset zone. (B) Difference in metric agreement between seizure onset zone-sparing and seizure onset zone-targeted subsampling. Each point represents the patient-specific difference in metric agreement between subsampling that spares the seizure onset zone electrode contacts and subsampling that targets the seizure onset zone electrode contacts. Positive values indicate that sparing the seizure onset zone from removal yields a higher agreement with the original network statistic than does targeting the seizure onset zone for removal. Horizontal lines show the average difference in metric agreement across patients. No difference in agreement was significantly different from 0 (two-sided one-sample *t* test, α = 0.05/8 for Bonferroni correction), signifying that metrics are equally vulnerable to subsampling that spares versus targets the seizure onset zone. However, there was nonsignificantly higher transitivity metric agreement when the seizure onset zone was spared. The horizontal position of individual points was determined by random jitter to improve readability.

There was no significant association between metric agreement and distance of the removed electrode contacts from the seizure onset zone for any metric (one-sample two-sided *t* test: control centrality, *t* = 0.80, *p* = 0.433; node strength, *t* = 1.25, *p* = 0.222; betweenness centrality, *t* = −0.95, *p* = 0.352; eigenvector centrality, *t* = 1.02, *p* = 0.318; clustering coefficient, *t* = 1.23, *p* = 0.230; synchronizability, *t* = −0.26, *p* = 0.793; global efficiency, *t* = 0.74, *p* = 0.469; transitivity, *t* = 0.37, *p* = 0.717). This pattern of findings implies that all metrics are equally sensitive to removing electrode contacts near versus distant from the seizure onset zone (Figure 3). This result was invariant to the choice of peri-ictal time window, choice of frequency band, choice of seizure, exclusion of non-ILAE 1 outcome patients, use of resection zone rather than seizure onset zone (excluding non-ILAE 1 patients), choice of network density, and choice of time window (Supplemental Table 4) as well as to the choice of removal percentage (Supplemental Table 5).

When we compare metric agreement removing *all* seizure onset zone electrode contacts as opposed to removing only *non*-seizure onset zone electrode contacts, there was again no significant difference in metric agreement between the seizure onset zone-sparing and seizure onset zone-targeted approach for any metric (paired two-sided *t* test: control centrality *t* = −0.99, *p* = 0.331; node strength, *t* = 0.84, *p* = 0.408; betweenness centrality, *t* = 1.43, *p* = 0.167; eigenvector centrality, *t* = 0.81, *p* = 0.424; clustering coefficient, *t* = 1.10, *p* = 0.283; synchronizability, *t* = 2.33, *p* = 0.028; global efficiency, *t* = 1.74, *p* = 0.095; transitivity, *t* = 2.65, *p* = 0.014; α = 0.00625, Bonferonni correction for eight metrics). These findings suggest that sparing versus targeting seizure onset zone electrode contacts for removal has equivalent effects on most network statistics. Across conditions, transitivity generally displayed the largest differences between seizure onset zone-sparing agreement and seizure onset zone-targeted agreement, although these differences were significant only for the EEC + 10-s condition, the second seizure, and 50% network density (Supplemental Table 6). To determine whether the trend in transitivity perturbation was to increase or decrease network transitivity, we obtained the signed relative difference (as opposed to the metric ageement, which is unsigned) between the subsampled and the original transitivity measure, and compared this for the seizure onset zone-sparing and seizure onset zone-targeted subsampling methods. The relative difference in transitivity was nonsignificantly higher (more positive) when seizure onset zone-targeted subsampling was performed, which persisted across all time periods, frequency bands, and the second seizure (Supplemental Table 6, two-sided paired *t* test, α = 0.00625, Bonferonni correction for eight metrics). This suggests that removing seizure onset zone electrode contacts may disproportionately *increase* transitivity (although this result was nonsignificant for most conditions).

When we used the approach calculating the percentage of seizure onset zone-sparing agreements larger than the seizure onset zone-targeted agreement, we found that the seizure onset zone-sparing agreement was significantly higher than the seizure onset zone-targeted agreement for both synchronizability (one sample two-sided *t* test, *t* = 3.72, *p* = 0.001) and transitivity (*t* = 3.04, *p* = 0.005) with no significant difference for other measures (Supplemental Table 6). The directions of these results were the same as those seen in the above analysis. However, given the dependence between seizure onset zone-sparing subsamples discussed in the Methods section, we believe that this analysis is less conservative than our primary analysis above.

### Jackknife Confidence Intervals

**Figure 4.**
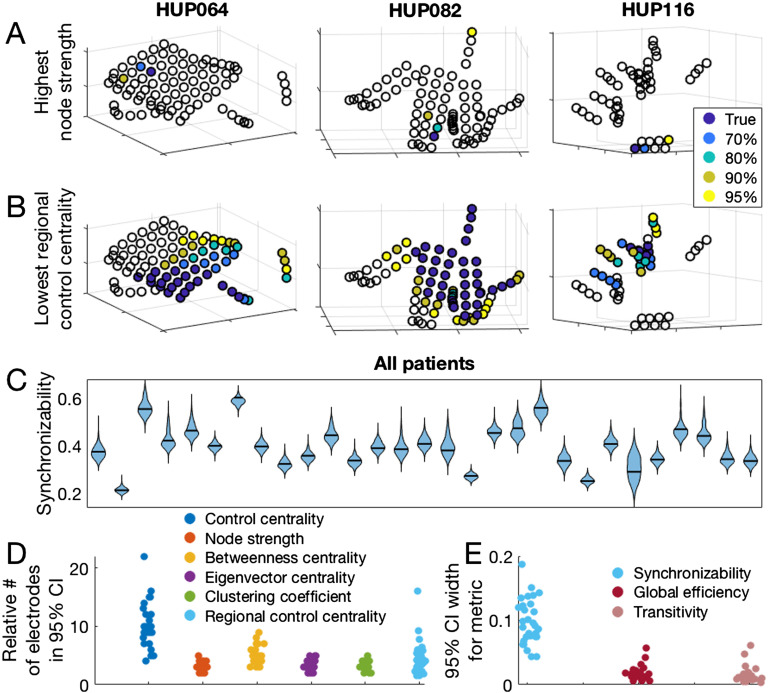
Jackknife subsampling to estimate confidence regarding network metric values. (A) The location of the electrode with the highest node strength, as well as the electrodes accounting for various percentages of highest node strength occurrences in 1,000 random jackknife subsampling networks for three example patients. (B) The location of the most synchronizing region (which is the region with the lowest regional control centrality), and the regions accounting for various percentages of the most synchronizing region occurrences in 1,000 random jackknife subsampling networks for three patients. (C) Patient-specific synchronizability distributions across subsamples. Each separate violin represents a patient and shows the distribution of synchronizability values obtained across 1,000 random jackknife subsamples. Horizontal black lines show the original value in the non-subsampled network. (D) Number of electrodes forming the 95% jackknife confidence electrode set for each nodal metric, for all patients. For each nodal metric, each dot shows the patient-specific number of electrodes accounting for 95% of all occurrences of the maximum (minimum for control centrality and regional control centrality) metric value in 1,000 random jackknife subsampling networks. For regional control centrality, this number is divided by the number of electrodes forming the minimum regional control centrality in the original non-subsampled network to obtain a ratio. (E) Width of the 95% jackknife confidence interval for each global metric, for all patients. For each global metric, each dot shows the patient-specific width of the 95% jackknife confidence interval of the metric value across 1,000 random subsamples. In D and E, the horizontal position of individual points was determined by random jitter to improve readability. This figure demonstrates the variability in confidence of network theory results across patients that can be revealed by jackknife subsampling.

Both nodal and global metrics varied across patients with respect to jackknife confidence intervals produced by subsampling (Figure 4A–C). The median and range for the number of electrode contacts accounting for 95% of all jackknife instances of the maximum nodal metric (minimum for control centrality) was 3 (range 2–5) for node strength, 4 (3–9) for betweenness centrality, 3 (2–5) for eigenvector centrality, 3 (2–5) for clustering coefficient, and 9 (4–22) for control centrality. The median number of electrode contacts accounting for 95% of all jackknife instances of the minimum regional control centrality (where the set of electrode contacts with minimum regional control centrality is the set, equal in number to the number of resected electrode contacts, that together produces the largest negative change in synchronizability when removed) was 48 (range 12–93). The median ratio between this number and the number of electrode contacts forming the true minimum regional control centrality set was 4.0 (range 1.6–16.0; Figure 4D). Regarding global metrics, the median width of the 95% jackknife confidence interval was 0.094 (range 0.045–0.192) for synchronizability, 0.016 (range 0.006–0.058) for global efficiency, and 0.012 (range 0.004–0.062) for transitivity (Figure 4E). These results demonstrate the heterogeneity among patients in the level of confidence in estimated network statistics that can be revealed by the jackknife algorithm. The locations of electrode contacts with the maximum or minimum metric values, as well as the results of jackknife subsampling, varied somewhat across time periods, choice of frequency band for coherence, seizure, network density, and time window for coherence calculations (Supplemental Figure 1, Supplemental Table 7).

## DISCUSSION

Handling missing data is a long-standing problem in science in general and is particularly problematic in network science, where a missing node may limit our understanding of the entire network (Guimerà & Sales-Pardo, 2009). In social networks, missing data can dramatically alter network statistics (Albert et al., 2000, 2004; Kossinets, 2006). In the field of neuroscience, Jalili demonstrated that global efficiency of scalp EEG-based functional networks in healthy individuals was highly sensitive to the removal of certain nodes (Jalili, 2015). To our knowledge, this is the first study examining the reliability of network statistics in the epileptic brain and in iEEG data. We determined that network measures differ in robustness to spatial subsampling, and that the sensitivity to sampling does not depend on the distance from the seizure onset zone. We also found that more extensive implants were more robust to subsampling. Finally, we developed and applied an algorithm using jackknife subsampling of electrode contacts to estimate confidence in nodal and global statistics in patient brain networks.

### Functional Network Metrics Exhibit Differential Reliability Under Spatial Subsampling

Metric reliability for all network measures decreased with a greater degree of missing data, which has previously been reported in social networks (Kossinets, 2006; Smith & Moody, 2013). Among examined nodal metrics, node strength and eigenvector centrality were most reliable and control centrality was least reliable; among the global metrics we tested, transitivity was most reliable and synchronizability was least reliable. The difference in reliability across metrics reflects, in part, the underlying sensitivity of each metric to graph topology. Prior studies in social networks have also observed that node strength is more robust to subsampling than betweenness centrality (Costenbader & Valente, 2003; Galaskiewicz, 1991; Smith & Moody, 2013). The relative robustness of node strength and eigenvector centrality compared with other nodal measures may reflect that metrics that primarily incorporate immediate connections to the node of interest are less sensitive to subsampling than metrics that more strongly weigh multistep connections. The preserved ordinality of network metric reliability across most patients, timescales, and frequency bands suggests that this result is generalizable. Clinically, applying network statistics that are more robust to spatial sampling may be preferable in cases in which the electrode coverage of important regions is uncertain. The ability of each metric to capture network behavior must be weighed against its spatial reliability if such personalized models are to be translated clinically.

Sensitivity to incomplete sampling depends somewhat on the number of electrode contacts forming the original implantation. Although synchronizability and clustering coefficient were the only global and nodal measures, respectively, to demonstrate a significant positive relationship between number of electrode contacts and metric reliability, all other measures except control centrality demonstrated nonsignificant positive relationships. This pattern of findings suggests that for most network measures, greater robustness can be achieved in part through more extensive electrode coverage. This agrees with work finding that more extensive electrode coverage results in better predictions of surgical outcome (Wang et al., 2019). Mechanistically, this may imply that random subsampling is less likely to remove important hubs in larger networks. Alternatively, perhaps information about missing nodes and edges can be inferred from the remaining components of the network, which is facilitated by a larger starting network. Clinically, this suggests that implanting a greater number of electrode contacts may increase our confidence in network statistics. This benefit would have to be weighed against the risks of more extensive coverage, including hemorrhage and infection (Mullin et al., 2016). Network metrics were generally more sensitive to contiguous removal than to random removal of electrodes. This may reflect spatial correlation of brain signals, such that information from a missing electrode contact may be more easily inferred from remaining neighbor contacts (Betzel et al., 2019; Lagarde et al., 2018). Alternatively, removing contiguous contacts may increase the probability of removing the entire set of critical electrode contacts that are needed to localize seizure generators. The analysis removing contiguous electrode contacts may better approximate the clinical scenario of leaving out an entire electrode or region from sampling, whereas the analysis removing random contacts better simulates choosing sparser network coverage. Consideration of which type of “missed coverage” applies in a given clinical context will inform how strongly that missing coverage may affect network results.

One limitation of the reliability measure as an approximation of metric robustness to subsampling is that nodal network metrics and their variances across subsampling are not normally distributed, and so the reliability measure may be disproportionately influenced by electrode contacts with more extreme values. The fact that nodal metrics had the same differential ordering in robustness to subsampling as measured by Spearman rank correlation between the original and subsampled metric supports the validity of the ordering of nodal metric reliabilities.

### Metric Sensitivity to Incomplete Sampling Is Independent of Distance From the Seizure Onset Zone

All metrics tested were equally sensitive to removing nodes in close or far proximity from the clinician-defined seizure onset zone. Most metrics were also equally sensitive to subsampling that targeted removal of the complete seizure onset zone and subsampling that spared the seizure onset zone. This may be because the seizure-generating network is a relatively small subset of the full peri-ictal network, and thus perturbation of the seizure-generating network has a small effect on the network as a whole. These results are practically concerning in the case of metrics with lower reliability to spatial subsampling, such as control centrality and synchronizability, because placement of electrodes in the network periphery away from clinical zones of interest is often variable across patients and across epilepsy centers. To increase the clinical confidence in the results of these network measures, the incomplete network may be supplemented using structural connectivity data and atlas-based approaches (Betzel et al., 2017; Fan, Li, Yu, & Jiang, 2016; Greicius, Supekar, Menon, & Dougherty, 2009; Liao et al., 2011; Reddy et al., 2018; Shah, Bassett, et al., 2018). Network theory also proposes several methods of predicting missing links (Guimerà & Sales-Pardo, 2009; Lü, Pan, Zhou, Zhang, & Stanley, 2015; Lü & Zhou, 2011; Pan, Zhou, Lü, & Hu, 2016). The finding of nonsignificantly increased sensitivity of the transitivity measure to removing the entire seizure onset zone, along with a disproportionate *increase* in transitivity when seizure onset zone electrodes are removed, may reflect a tendency of nodes in the seizure onset zone to form widespread network connections peri-ictally. Practically, this finding also suggests that transitivity—and more specifically, subsampling-induced change in transitivity—is a promising measure to identify electrodes overlying the seizure onset zone. Of note, the seizure onset zone-targeted versus sparing subsampling method introduces a potential bias in that removing the seizure onset zone as opposed to random non-seizure onset zone electrodes disproportionately targets electrodes that are spatially clustered together. If anything, we would expect this bias to act to decrease transitivity when seizure onset zone electrodes are removed, as we might expect electrodes in close proximity to have a higher within-group clustering and thus increase the overall transitivity of the network.

A limitation of this analysis is that clinical methods for identifying the seizure onset zone are imperfect, and so the clinician-defined seizure onset zone may not capture actual seizure generators. When redefining the hypothesized site of seizure generation as the resection zone (for good-outcome patients) we also found no difference in removing nodes in close or distant proximity from the resection zone. However, it is possible that neither method accurately captured true seizure generators.

### Jackknife Network Subsampling Generates Confidence Intervals for Virtual Resection

Prior work has used global and nodal network measures to stratify surgical candidates and to select nodes for resection (Lopes et al., 2017; Shah, Bernabei, et al., 2018; Sinha et al., 2017; Tomlinson, Porter, & Marsh, 2017). Here we provide a simple algorithm to augment these methods by determining patient-specific confidence in the robustness of estimated statistics to small perturbations in spatial sampling. Similar resampling-based approaches have been used in social networks (Duval, Christensen, & Spahiu, 2010; Lusseau, Whitehead, & Gero, 2008; Ohara, Saito, Kimura, & Motoda, 2014), in gene expression data (de Matos Simoes & Emmert-Streib, 2012), and in resting fMRI data of healthy subjects (Cheng et al., 2017). The heterogeneity in confidence across patients may be used to stratify patients into those for whom enough information of the epileptic network is captured to accurately use personalized network models and those for whom models are likely to be inaccurate because of implantation strategy. In this study we do not claim to identify the ideal network model among the many published studies. The jackknife subsampling method may be easily applied to any of these network models.

### Methodological Limitations and Future Directions

Our method of network subsampling can examine how networks change only with the removal of electrode contacts, and not upon the addition of contacts. For an intracranial EEG study that has parsimoniously captured seizure onset and spread, using our statistical subsampling method may miss critical electrode contacts and thus erroneously characterize the network as unreliable. However, for those patients who have very clear seizure onset and propagation, personalized network models of epilepsy may not be required to guide surgical planning. An additional limitation is that our measure of nodal metric similarity—the Spearman rank correlation—may not capture how network model predictions practically change with subsampling. It is conceivable that a network metric would demonstrate low reliability to subsampling as defined in this paper, but still generate consistent predictions about the optimal site of surgical intervention depending on how these predictions are formed. Different groups have proposed different methods to direct surgical targeting using network statistics (Kini et al., 2019; Proix et al., 2018; Shah, Bernabei, et al., 2018; Sinha et al., 2017, 2019), and our proposed subsampling method can be used to test the sensitivity of any targeting predictions to incomplete spatial sampling, similar to the jackknife procedure described in the section above.

The jackknife analysis results varied somewhat across time, frequency band, and choice of seizure, reflecting the different states of the network. These observations underscore the fact that spatial sampling is one of several sources of bias of the network statistics. Further exploration of the sensitivity of network statistics to the choice of seizure, time, and frequency band will be necessary for useful clinical implementation. Also, additional steps toward translating this work into clinical care will require expanding our dataset to include larger numbers of patients, stereo EEG implantations, and those treated with focal laser ablation and perhaps brain stimulation devices.

While in this study we implemented only data-driven metrics that describe underlying network properties, there is significant interest in fitting generative models of neural population dynamics to brain networks. One such example, the Epileptor model (Jirsa, Stacey, Quilichini, Ivanov, & Bernard, 2014), is a neural mass model that describes many relevant epileptic dynamics and is currently under study as a clinical trial in Europe to guide epilepsy surgery (Proix et al., 2018). Our network subsampling approach may also be used for generative neurophysiologic models and gives confidence to their clinical utility.

### Conclusions

The field of network science provides a promising set of tools for understanding epilepsy dynamics and for surgical planning. However, the robustness of empirical estimates of network statistics to incomplete electrode sampling is not well understood. We have shown variability across network measures in robustness to incomplete sampling. Network measures are equally vulnerable to removing electrode contacts near versus distant from the seizure onset zone, and to removing electrode contacts within the seizure onset zone versus an equivalent number of non-seizure onset zone electrode contacts. Robustness to incomplete sampling is highly heterogeneous across patients, and jackknife subsampling is a simple algorithm to obtain patient-specific confidence in the results of network statistics. The choice of individual network models should be based upon the intended application and on the clinical certainty that important seizure generators have been sampled by intracranial implants.

## DATA AVAILABILITY

All EEG records and annotations are available on the International Epilepsy Electrophysiology Portal (https://www.ieeg.org; Kini, Davis, & Wagenaar, 2016; Wagenaar et al., 2013). All code is available on GitHub (https://github.com/erinconrad/network-subsets; Conrad & Bernabei, 2019). We include an example script and an associated example dataset in a single patient to demonstrate usage of the code base. We do not publicly host network adjacency matrices because of file size, but will share them with readers upon request.

## SUPPORTING INFORMATION

Supporting information for this article is available at https://doi.org/10.1162/netn_a_00131.

## AUTHOR CONTRIBUTIONS

Erin Conrad: Conceptualization; Formal analysis; Methodology; Writing - Original Draft. John Bernabei: Conceptualization; Formal analysis; Methodology; Writing - Original Draft; Writing - Review & Editing. Lohith Kini: Data curation; Formal analysis; Writing - Review & Editing. Preya Shah: Conceptualization; Writing - Review & Editing. Fadi Mikhail: Data curation. Ammar Kheder: Data curation. Russell Shinohara: Formal analysis; Writing - Review & Editing. Kathryn Davis: Conceptualization; Supervision; Writing - Review & Editing. Danielle Bassett: Conceptualization; Supervision; Writing - Review & Editing. Brian Litt: Supervision; Writing - Review & Editing.

## FUNDING INFORMATION

Brian Litt, National Institute of Neurological Disorders and Stroke (http://dx.doi.org/10.13039/100000065), Award ID: R01-NS099348-01. Kathryn Davis, National Institute of Neurological Disorders and Stroke (http://dx.doi.org/10.13039/100000065), Award ID: R01-NS099348-01. Danielle Bassett, National Institute of Neurological Disorders and Stroke (http://dx.doi.org/10.13039/100000065), Award ID: R01-NS099348-01. Brian Litt, National Institute of Neurological Disorders and Stroke (http://dx.doi.org/10.13039/100000065), Award ID: 1-T32-NS-091006-01. Fadi Mikhail, National Institute of Neurological Disorders and Stroke (http://dx.doi.org/10.13039/100000065), Award ID: 1-T32-NS-091006-01. Erin Conrad, National Institute of Neurological Disorders and Stroke (http://dx.doi.org/10.13039/100000065), Award ID: R25-NS065745. Kathryn Davis, National Institute of Neurological Disorders and Stroke (http://dx.doi.org/10.13039/100000065), Award ID: K23 NS073801. Brian Litt, Mirowski Family Foundation. Brian Litt, Neil and Barbara Smit. Brian Litt, Jonathan Rothberg. Kathryn Davis, Thornton Foundation. Danielle Bassett, Alfred P. Sloan Foundation (http://dx.doi.org/10.13039/100000879). Danielle Bassett, John D. and Catherine T. MacArthur Foundation (http://dx.doi.org/10.13039/100000870). Russell Shinohara, National Institutes of Health (http://dx.doi.org/10.13039/100000002), Award ID: R01MH112845. Russell Shinohara, National Institutes of Health (http://dx.doi.org/10.13039/100000002), Award ID: R01NS060910. Danielle Bassett, ISI Foundation. Russell Shinohara, National Multiple Sclerosis Society. Russell Shinohara, Race to Erase MS. Fadi Mikhail, the Institute of Translational Medicine and Therapeutics (ITMAT) of the Perelman School of Medicine at the University of Pennsylvania.

## COMPETING INTERESTS

Erin Conrad has received consulting income from Ceribell. Russell Shinohara has received consulting income and has served on a scientific advisory board for Genentech/Roche, and has received income for editorial/reviewership duties from the American Medical Association and Research Square.

## TECHNICAL TERMS

Network node: A discrete region in a network, connected to other regions (nodes) via edges.
Node strength: A nodal metric that represents the sum of the weights of the connections to the node of interest.
Eigenvector centrality: A nodal metric that represents the sum of connection weights to the node of interest, weighted by the eigenvector centrality of the connected nodes.
Betweenness centrality: A nodal metric calculating the fraction of all shortest paths in a network that pass through a node of interest.
Clustering coefficient: A nodal metric that measures the tendency of a node to cluster into tight groups.
Control centrality: A nodal metric that represents the change in synchronizability that occurs with removal of a node of interest.
Global metric: Metrics in network neuroscience representing a property of the overall network.
Global efficiency: A global metric that is thought to represent how easily information is transmitted through a network.
Synchronizability: A global metric that quantifies the stability of the fully synchronous network state.
Transitivity: A global metric that is thought to represent the degree to which nodes in a graph tend to cluster together.
Intracranial EEG: Electroencephalogram (EEG) in which electrodes are placed inside the skull, either on top of the brain surface (grid and strip electrodes) or in the brain (depth electrodes).
Reliability: A statistical measure of the consistency of a metric, defined as the variance of the true metric divided by the total variance, where the total variance is the sum of the variance of the true metric and the variance of the error.
Jackknife subsampling: A subsampling technique (also called jackknife resampling) in which a random subset of observations is removed and this is repeated many times in order to obtain estimates of sample variance.
EEC: Earliest electrographic change, the first appreciable EEG change during a seizure.
Functional coherence: A method of constructing functional networks that estimates the correlation between two electrode contact signals in the frequency domain.
Nodal metrics: Metrics in network neuroscience measuring the importance of individual nodes.
