# Supplementary material for: The sensitivity of network statistics to incomplete electrode sampling on intracranial EEG
Source: Netw Neurosci. 2020 May 1;4(2):484–506. doi: 10.1162/netn_a_00131 (PMC7286312; doi:10.1162/netn_a_00131)
Supplement: Supplementary file 1 [file netn-04-484-s001.pdf]

Conrad, E. C., Bernabei, J. M., Kini, L. G., Shah, P., Mikhail, F., Kheder, A., Shinohara, R. T., Davis, K. A., Bassett, D. S., & Litt, B. (2020). Supporting information for "The sensitivity of network statistics to incomplete electrode sampling on intracranial EEG." *Network Neuroscience*, *4*(2) 484–506. https://doi.org/10.1162/netn_a_00131

Supplemental Materials:

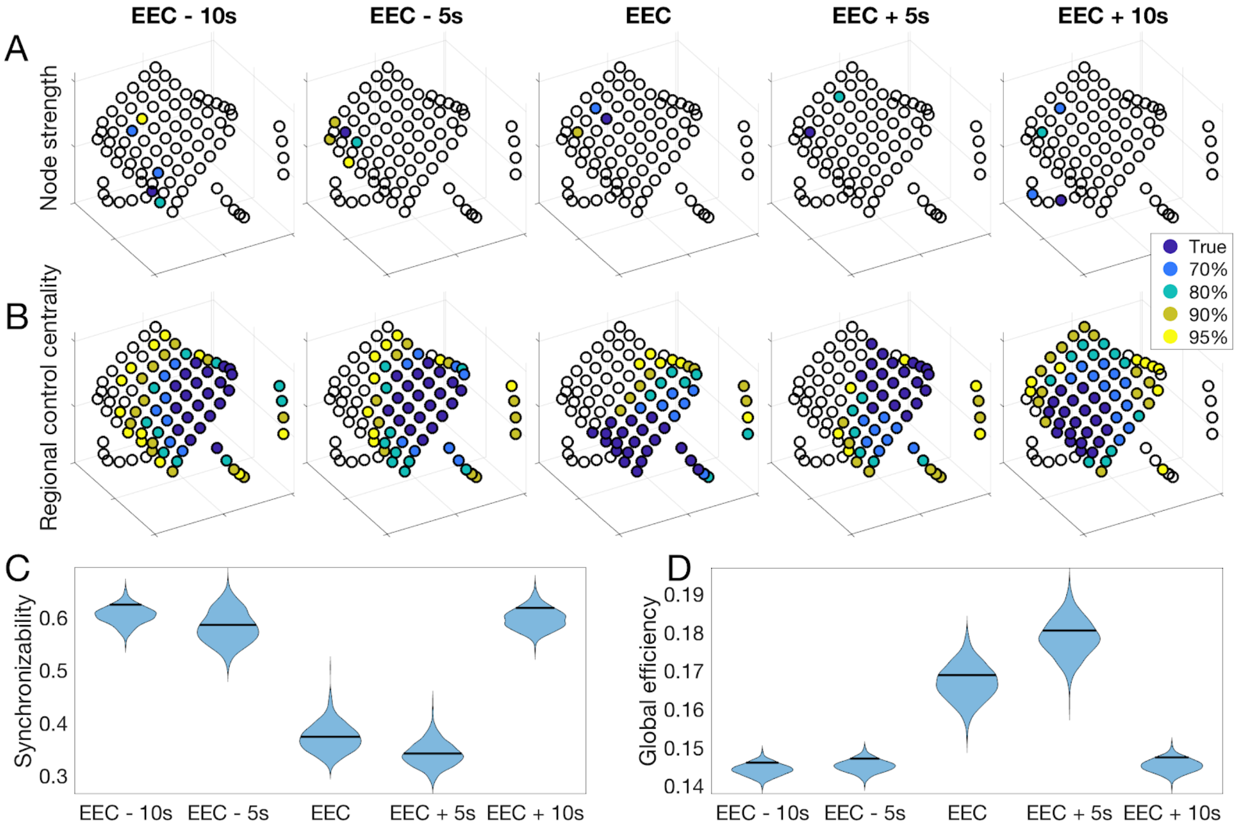

Supplemental Figure 1: **Time dependence of jackknife subsampling results**. All data shown is for a single example patient (HUP064). A: The location of the electrode with the highest node strength, as well as the electrodes accounting for various percentages of highest node strength occurrences in 1,000 random jackknife subsampling networks at five different time periods. B: The location of the most synchronizing region, and the regions accounting for various percentages of the most synchronizing region occurrences in 1,000 random jackknife subsampling networks at five different time periods. C: The distribution of synchronizability values obtained with 1,000 random jackknife subsamplings for each time period. Horizontal lines denote synchronizability in the original non-subsampled network. D: The distribution of global efficiency values obtained with 1,000 random subsamplings for each time period. Horizontal lines denote global efficiency in the original non-subsampled network.

Patient ID	Sex	Age at onset	Age at surgery	Localization	Pathology	ILAE outcome	Grids	Strips	Depths
HUP064	M	3	22	LFL	MCD	1	64	22	0
HUP065	M	2	36	RTL	MCD	1	64	0	0

Conrad, E. C., Bernabei, J. M., Kini, L. G., Shah, P., Mikhail, F., Kheder, A., Shinohara, R. T., Davis, K. A., Bassett, D. S., & Litt, B. (2020). Supporting information for “The sensitivity of network statistics to incomplete electrode sampling on intracranial EEG.” *Network Neuroscience*. Advance publication. https://doi.org/10.1162/netn_a_00131

HUP068	F	13	28	RTL	HS/MTS	1	63	20	0
HUP070	M	12	33	LFPL	MCD	2	63	0	0
HUP073	M	5	40	RFL	MCD	1	0	52	0
HUP074	F	5	25	LTL	MCD	1	63	29	22
HUP075	F	52	57	LTL	HS/MTS	5	58	34	14
HUP078	M	2 mo	54	LTL	HS/MTS	4	63	24	14
HUP080	F	35	41	LTL	Gliosis	2	62	18	16
HUP082	F	34	56	RTL	HS/MTS	1	64	14	8
HUP083	M	8	29	LPL	Gliosis	2	59	22	0
HUP086	F	18	25	LTL	Gliosis	1	60	32	8
HUP087	M	19	24	LFL	Tumor/Vascular/Infection	3	60	12	12
HUP088	M	13 mo	24	LFL	HS/MTS	1	0	43	11
HUP094	F	20	48	RTL	Gliosis	2	0	64	20
HUP105	M	27	39	RTL	Tumor/Vascular/Infection	1	0	43	12
HUP106	F	24	45	LTL	HS/MTS	2	64	36	16
HUP107	M	5	36	RTL	HS/MTS	1	64	38	16
HUP111A	F	28	40	RTL	HS/MTS	1	0	32	16
HUP111B	F	28	40	RTL	HS/MTS	1	47	42	12
HUP116	F	51	58	RTL	unknown	1	0	0	50
Study012	M	23	37	RFL	Gliosis	1	57	22	0
Study016	F	5	36	RFTL	Gliosis	4	47	14	0
Study017	M	unknown	unknown	RTL	unknown	4	0	0	16
Study019	M	31	33	LTL	Gliosis	5	60	28	8
Study020	M	5	10	RFL	Gliosis	4	40	16	0
Study022	F	11-20	21-30	LTL	Gliosis	5	42	12	0
Study028	M	4	5	LFPL	Gliosis	4	64	5	0
Study029	F	unknown	unknown	RTL	Gliosis	5	23	30	8

Supplemental Table 1. **Clinical and electrode information.** 2-year post-surgical outcomes were based on the International League Against Epilepsy (ILAE) classification system (class 1-6). HUP: Hospital of

the University of Pennsylvania, M: male, F: female, mo: months, LFL: left frontal lobe, LFPL: left frontoparietal lobe, LTL: left temporal lobe, RTL: right temporal lobe, RFTL: right frontotemporal lobe, RFL: right frontal lobe, MCD: malformation of cortical development; FCD: focal cortical dysplasia, HS/MTS: hippocampal sclerosis/mesial temporal sclerosis.

	EEC - 10 s	EEC - 5 s	EEC	EEC + 5 s	EEC + 10 s	Beta	Contiguous removal	Seizure #2	Last seizure	Density 50%	2 s window
Control centrality	0.80	0.82	0.84	0.79	0.81	0.80	0.82	0.79	0.80	0.73	0.82
Node strength	0.95	0.95	0.96	0.96	0.96	0.95	0.95	0.96	0.95	0.96	0.95
Betweenness centrality	0.88	0.89	0.88	0.89	0.88	0.88	0.88	0.89	0.88	0.89	0.90
Eigenvector centrality	0.95	0.95	0.97	0.96	0.96	0.95	0.94	0.96	0.95	0.97	0.95
Clustering coefficient	0.94	0.94	0.95	0.95	0.95	0.94	0.91	0.95	0.94	0.90	0.94
Synchronizability	0.95	0.93	0.92	0.93	0.94	0.91	0.85	0.94	0.96	0.94	0.95
Global efficiency	0.98	0.97	0.98	0.99	0.99	0.98	0.92	0.98	0.99	0.98	0.98
Transitivity	0.98	0.97	0.98	0.99	0.99	0.98	0.93	0.97	0.99	0.98	0.98

Color scale: 0.7, 0.75, 0.8, 0.85, 0.9, 0.95, 1

Supplemental Table 2. **Metric reliability for alternative conditions.** The metric reliability, defined in the text, for alternative conditions. The range of reliabilities shown is 0.7 (dark blue) to 1 (bright yellow). Default conditions are for the earliest electrographic change (EEC), high gamma frequency, the first seizure, random electrode removal, an unthresholded network, a one-second time window for calculating coherence, and a 20% removal percentage (column 3 shows these default conditions, which are also reported as the primary results in the paper). Unless otherwise specified, each column uses the default conditions. The different columns are: different time periods relative to the EEC (columns 1-5), beta frequency coherence as opposed to high gamma frequency coherence (column 6), removal of a contiguous set of electrodes as opposed to random set (column 7), the second seizure and last seizure as opposed to the first seizure (column 8-9), a thresholded network to achieve a density of 50% (column 10), and a two-second time window for coherence calculations, as opposed to a one-second window (column 11).

Conrad, E. C., Bernabei, J. M., Kini, L. G., Shah, P., Mikhail, F., Kheder, A., Shinohara, R. T., Davis, K. A., Bassett, D. S., & Litt, B. (2020). Supporting information for "The sensitivity of network statistics to incomplete electrode sampling on intracranial EEG." *Network Neuroscience*. Advance publication. https://doi.org/10.1162/netn_a_00131

	EEC, 80%	EEC, 60%	EEC, 40%	EEC, 20%
Control centrality	0.62	0.70	0.76	0.84
Node strength	0.72	0.84	0.92	0.96
Betweenness centrality	0.62	0.70	0.79	0.88
Eigenvector centrality	0.75	0.86	0.92	0.97
Clustering coefficient	0.60	0.78	0.88	0.95
Synchronizability	0.65	0.77	0.85	0.92
Global efficiency	0.82	0.91	0.95	0.98
Transitivity	0.84	0.91	0.95	0.98

Supplemental Table 3. **Metric reliability for all removal percentages.** The metric reliability, defined in the text, for all removal percentages tested. The range of reliabilities displayed is 0.6 (dark blue) to 1 (bright yellow). All data is shown for the time period at the EEC, high gamma coherence, and random electrode contact removal. The fourth column shows the 20% removal condition, which is the primary analysis reported in the paper.

Conrad, E. C., Bernabei, J. M., Kini, L. G., Shah, P., Mikhail, F., Kheder, A., Shinohara, R. T., Davis, K. A., Bassett, D. S., & Litt, B. (2020). Supporting information for "The sensitivity of network statistics to incomplete electrode sampling on intracranial EEG." *Network Neuroscience*. Advance publication. https://doi.org/10.1162/netn_a_00131

	EEC - 10 s	EEC - 5 s	EEC	EEC + 5 s	EEC + 10 s	Beta	Seizure #2	Last seizure	Density 50%	ILAE 1 only	Resection zone	2 s window
Control centrality	-0.09	0.02	0.80	2.64	1.15	0.49	1.06	1.32	0.78	1.81	1.69	0.67
Node strength	1.47	0.07	1.25	1.30	-0.83	1.76	0.95	0.21	0.70	0.71	1.84	2.18
Betweenness centrality	-0.83	-0.22	-0.95	-1.22	0.13	0.04	-0.31	-0.33	-0.97	-0.03	-1.70	0.22
Eigenvector centrality	1.60	0.22	1.02	1.72	-0.47	1.92	1.37	-0.40	1.00	0.82	1.91	1.48
Clustering coefficient	1.75	0.02	1.23	1.53	-0.84	1.16	1.22	0.02	1.99	0.60	1.68	2.18
Synchronizability	-0.30	0.80	-0.26	0.85	0.84	-0.11	-1.06	0.59	0.43	-0.08	0.66	1.31
Global efficiency	2.00	0.79	0.74	0.54	2.31	1.67	-0.61	1.25	0.96	0.43	1.78	0.74
Transitivity	-0.02	1.43	0.37	0.30	1.70	0.55	-1.39	1.34	-0.24	-0.22	0.52	0.05

Supplemental Table 4. **Association between metric agreement and distance of removed electrodes from seizure onset zone for alternative conditions.** Values denote the *t*-statistic evaluating the patient-aggregated Fisher's transformed Spearman rank correlations for the distance-agreement associations. The range of values displayed is from -4 (dark blue) to 4 (bright yellow) (the display range chosen is larger than the actual range of values so as to highlight only highly significant values). The method for calculating the agreement-distance association is described in the text. Positive values indicate that the metric is more sensitive to removing electrodes near the resection zone. Default conditions are for the earliest electrographic change (EEC), high gamma frequency, the first seizure, random electrode removal, an unthresholded network, a one-second time window for calculating coherence, and a 20% removal percentage (column 3 shows these default conditions, which are also reported as the primary results in the paper). Unless otherwise specified, each column uses the default conditions. The different columns are: different time periods relative to the EEC (columns 1-5), beta frequency coherence (column 6), the second seizure and last seizure (columns 7-8), a thresholded network to achieve a density of 50% (column 9), excluding any patient for whom the ILAE outcome was not 1 (column 10), utilizing the resection zone as opposed to the seizure onset zone as the hypothesized location of seizure generation, while excluding non-ILAE 1 patients (column 11), and a two-second time window for coherence calculations, as opposed to a one-second window (column 12). No association was significant for $\alpha = 0.05/8$ (Bonferroni correction).

Conrad, E. C., Bernabei, J. M., Kini, L. G., Shah, P., Mikhail, F., Kheder, A., Shinohara, R. T., Davis, K. A., Bassett, D. S., & Litt, B. (2020). Supporting information for "The sensitivity of network statistics to incomplete electrode sampling on intracranial EEG." *Network Neuroscience*. Advance publication. https://doi.org/10.1162/netn_a_00131

	EEC, 80%	EEC, 60%	EEC, 40%	EEC, 20%
Control centrality	-0.58	0.61	2.23	0.80
Node strength	0.42	1.53	1.53	1.25
Betweenness centrality	0.74	0.16	0.49	-0.95
Eigenvector centrality	0.47	1.72	1.46	1.02
Clustering coefficient	0.55	1.46	1.44	1.23
Synchronizability	1.28	2.25	0.95	-0.26
Global efficiency	0.34	0.89	0.90	0.74
Transitivity	0.54	0.54	0.55	0.37

Colour scale: 4, 3, 2, 1, 0, -1, -2, -3, -4

Supplemental Table 5. **Association between metric agreement and distance of removed electrodes from seizure onset zone for alternative removal percentages.** Values denote the *t*-statistic evaluating the patient-aggregated Fisher's transformed Spearman rank correlations for the distance-agreement associations. The method for calculating the agreement-distance association is described in the text. Positive values indicate that the metric is more sensitive to removing electrodes near the resection zone. The range of *t*-statistics shown is -4 (dark blue) to 4 (bright yellow). Values are shown for different percentages of removed electrodes. All results are for the time period at the earliest electrographic change (EEC), high gamma frequency coherence, and the first seizure. No association was significant for $\alpha = 0.05/8$ (Bonferroni correction). The fourth column shows the 20% removal condition, which is the primary analysis reported in the paper.

Conrad, E. C., Bernabei, J. M., Kini, L. G., Shah, P., Mikhail, F., Kheder, A., Shinohara, R. T., Davis, K. A., Bassett, D. S., & Litt, B. (2020). Supporting information for "The sensitivity of network statistics to incomplete electrode sampling on intracranial EEG." *Network Neuroscience*. Advance publication. https://doi.org/10.1162/netn_a_00131

	EEC - 10 s	EEC - 5 s	EEC	EEC + 5 s	EEC + 10 s	Beta	Seizure #2	Last seizure	Density 50%	ILAE 1 only	Resection zone	2 s window	Distribution
Control centrality	1.25	-0.35	-0.99	0.88	1.00	0.79	1.36	0.01	0.70	-0.01	1.35	0.80	0.80
Node strength	0.43	0.53	0.84	0.94	-1.07	1.12	0.87	1.37	0.84	-0.75	2.06	1.07	2.12
Betweenness centrality	-0.94	-1.43	1.43	0.38	0.10	-1.78	-0.35	-0.53	1.42	1.52	-1.13	0.61	2.19
Eigenvector centrality	0.22	0.12	0.81	0.87	-1.74	0.34	0.65	1.34	0.58	-0.27	2.15	0.93	1.33
Clustering coefficient	0.15	0.87	1.10	1.38	-1.54	1.36	0.77	1.67	0.73	0.31	1.90	1.44	2.82
Synchronizability	-0.21	0.63	2.33	0.86	1.57	1.08	1.60	2.33	1.00	1.85	1.01	1.25	3.72**
Global efficiency	2.53	1.13	1.74	0.74	2.88	1.61	2.23	2.65	1.45	0.32	0.63	1.31	1.49
Transitivity	2.63	2.08	2.65	2.97	3.08*	2.69	3.07*	1.95	3.49*	1.34	-0.43	2.66	3.04*
Transitivity signed	-1.71	-1.97	-0.70	-1.41	-2.39*	-1.01	-1.50	0.37	-0.08	-0.56	1.57	-2.11*	-0.43

Supplemental Table 6. **Difference in metric agreement between seizure onset zone-sparing subsampling and seizure onset zone-targeted subsampling.** Values denote the *t*-statistic for a paired *t*-test evaluating the patient-aggregated original-subsampled metric agreements when subsampling spares the seizure onset zone versus targets the seizure onset zone. The method for calculating metric agreement is described in the text. Positive values indicate that subsampling that targets the seizure onset zone perturbs the network metric more than subsampling that spares the seizure onset zone. Default conditions are for the earliest electrographic change (EEC), high gamma frequency, the first seizure, random electrode removal, an unthresholded network, a one-second time window for calculating coherence, and a 20% removal percentage (column 3 shows these default conditions, which are also reported as the primary results in the paper). Unless otherwise specified, each column uses the default conditions. The different columns are: different time periods relative to the EEC (columns 1-5), beta frequency coherence (column 6), the second seizure and last seizure (columns 7-8), a thresholded network to achieve a density of 50% (column 9), excluding any patient for whom the ILAE outcome was not 1 (column 10), utilizing the resection zone as opposed to the seizure onset zone as the hypothesized location of seizure generation, while excluding non-ILAE 1 patients (column 11), a two-second time window for coherence calculations, as opposed to a one-second window (column 12), and an alternate analysis in which we compare the seizure onset zone-targeted agreement to the *distribution* of seizure onset zone-sparing agreements, rather than the mean seizure onset zone-sparing agreement, described in the Methods (column 13). The bottom row shows the *t*-statistics for the same test but comparing the *signed* relative difference in transitivity between the original and subsampled network. In this case, negative values indicate that removing - as opposed to

sparing - seizure onset zone electrodes has a greater tendency to *increase* transitivity relative to the original network. Asterisks indicate values that are significant for α = 0.05/8, and double asterisks indicate significant values for α = 0.01/8 (Bonferroni correction). There were lower transitivity agreements when the seizure onset zone electrode contacts were removed (but this was non-significant across most conditions).

	**EEC - 10 s**	**EEC - 5 s**	**EEC**	**EEC + 5 s**	**EEC + 10 s**	**Beta**	**Contiguous removal**	**Seizure #2**	**Last seizure**	**Density 50%**	**2 s window**
Node strength	3	3	3	3	3	4	3	3	4	3	3
Betweenness centrality	4	4	4	5	5	5	4	4	4	4	4
Eigenvector centrality	3	4	3	3	3	4	3	3	4	3	3
Clustering coefficient	3	3	3	3	3	4	3	3	3.5	5	3
Control centrality	9.5	11	9	8	7	10	6	9	9.5	11	11
Regional control centrality	52	51	48	51	47.5	52	50	44	52	55	49
Synchronizability	0.09	0.10	0.09	0.09	0.09	0.09	0.13	0.09	0.12	0.09	0.10
Global efficiency	0.01	0.02	0.02	0.02	0.02	0.01	0.02	0.02	0.02	0.02	0.02
Transitivity	0.01	0.01	0.01	0.01	0.01	0.01	0.02	0.01	0.01	0.03	0.01

Supplemental Table 7. **Results of jackknife subsampling method for alternative conditions.** Each nodal value shows the average number of electrodes accounting for 95% of all occurrences of the maximum metric value (minimum for control centrality and regional control centrality) across 1,000 jackknife subsamples. Each global value shows the width of the 95% jackknife confidence interval of the network metric across 1,000 jackknife subsamples. Default conditions are for the earliest electrographic change (EEC), high gamma frequency, the first seizure, random electrode removal, an unthresholded network, a one-second time window for calculating coherence, and a 20% removal percentage (column 3 shows these default conditions, which are also reported as the primary results in the paper). Unless otherwise specified, each column uses the default conditions. The different columns are: different time periods relative to the EEC (columns 1-5), beta frequency coherence as opposed to high gamma frequency coherence (column 6), removal of a contiguous set of electrodes as opposed to random set (column 7), the second seizure and last seizure as opposed to the first seizure (column 8-9), a thresholded network to achieve a density of 50% (column 10), and a two-second time window for coherence calculations, as opposed to a one-second window (column 11).
